# Legal Notes for Institutional Workers

**Published:** 1916-04-22

**Authors:** 


### LEGAL NOTES FOR INSTITUTIONAL WORKERS.

### Medical Officers and Old-Age Pensioners.

At the time the verdict was given we referred fully to the action by Dr. Wallace James, Haddington, for damages in respect of slander said to be contained in a letter written by a prominent local lady to the Chairman of Haddington Parish Council representing that the plaintiff, who is medical officer of the parish, had been guilty of gross and wilful failure of professional duty in omitting to procure the services of the district nurse to a patient. The jury returned a verdict for plaintiff of £1,000 damages, but that has now been considered excessive by the First Division and a new trial ordered. Probably the amount of damages will be adjusted between the parties, and on that point the case is of comparatively small importance. It, however, decides a question cf privilege which is of general interest to medical men in official positions. It was urged on defendant's behalf that the facts proved at the trial clearly disclosed that the occasion was privileged; and inasmuch as there was no evidence of any particular facts and circumstances inferring malice, the defendant claimed the verdict. The direction which Lord Anderson, who presided at the trial, was asked to give to the jury was as follows: "In respect that Mrs. Haldane, as an old-age pensioner, was a person to whom the Parish Council had a duty to see that she got medical relief when needed, the letter complained of was privileged." This direction Lord Anderson refused to give. The Division were of opinion that Lord Anderson was right. It was clear that the mere fact that the patient was an old-age pensioner in need of medical relief did not render it the duty of the plaintiff, as medical officer of the parish, to attend her. To entitle her to medical relief from the plaintiff it was necessary that relief should have been asked on her behalf, that her circumstances should have been inquired into by the parish authorities, and that permission should have been given. Until all that had taken place the plaintiff owed no duty to the patient in his capacity as medical officer of the parish. The refusal of the presiding judge to give that direction being thus approved, a novel and interesting argument was next submitted to the effect that the occasion was privileged, because the facts as they appeared to the defender at the time disclosed a case of privilege. That argument was considered unsound in law, and in the present case destitute of foundation in fact. The letter was addressed to the Chairman of the Parish Council under a misapprehension in fact, and was consequently not protected. The contention advanced for the defendant was that if she had plausible grounds for thinking that the pursuer did owe a duty to the patient in his capacity as medical officer, and if she honestly did so think, then the Court must hold the occasion privileged. The Court were unable to see how an occasion which in point of fact was not privileged could become privileged because the defendant in good faith and on grounds which commended themselves to her considered that it was privileged. Her good faith, however strong its foundation, could not convert a non-privileged into a privileged occasion.

### Bequests to Institutions.

A principle of law in which hospitals and benevolent institutions generally have a practical interest is that which makes it competent for a testator to benefit classes of persons or classes of objects, and leave to his trustees the task of selecting the particular objects to receive the benefit. When this principle is disputed, as it was in the recent House of Lords case of *Wordie's Trustees*, the ground usually taken up by relatives who would benefit were the bequest set aside is that it is void from uncertainty; but the judgment of the Court in this last case in no way weakens the rule that bequests to charitable institutions to be selected by executors is competent and will receive legal effect.

### Workmen's Compensation and Post-Mortems.

Under the Workmen's Compensation Act the employers must be notified of the accident, but want of such notice does not bar a claim by the workman or his representatives, unless the employer can show that he has been prejudiced in his defence by the want of notice. In a case lately decided we observe that the point of prejudice set up by the defendant employer was that the delay in giving notice of the accident—a fatal one—prevented him having a *post-mortem* examination made. The Court in the circumstances did not consider that sufficient prejudice to stop the claim for the workman's representatives.

